# DoSurvive: A webtool for investigating the prognostic power of a single or combined cancer biomarker

**DOI:** 10.1016/j.isci.2023.107269

**Published:** 2023-07-04

**Authors:** Hao-Wei Wu, Jian-De Wu, Yen-Ping Yeh, Timothy H. Wu, Chi-Hong Chao, Weijing Wang, Ting-Wen Chen

**Affiliations:** 1 Institute of Bioinformatics and Systems Biology, National Yang Ming Chiao Tung University, Hsinchu 30068, Taiwan; 2 Institute of Ecology and Evolutionary Biology, National Taiwan University, Taipei 10617, Taiwan; 3 Institute of Molecular Medicine and Bioengineering, National Yang Ming Chiao Tung University, Hsinchu 30068, Taiwan; 4 Department of Biological Science and Technology, National Yang Ming Chiao Tung University, Hsinchu 30068, Taiwan; 5 Center For Intelligent Drug Systems and Smart Bio-devices (IDS^2^B), National Yang Ming Chiao Tung University, Hsinchu 30068, Taiwan; 6 Institute of Statistics, National Yang Ming Chiao Tung University, Hsinchu 30068, Taiwan

**Keywords:** Epigenetics, Cancer systems biology, Proteomics, Transcriptomics

## Abstract

We present DoSurvive, a user-friendly survival analysis web tool and a cancer prognostic biomarker centered database. DoSurvive is the first database that allows users to perform multivariant survival analysis for cancers with customized gene/patient list. DoSurvive offers three survival analysis methods, Log rank test, Cox regression and accelerated failure time model (AFT), for users to analyze five types of quantitative features (mRNA, miRNA, lncRNA, protein and methylation of CpG islands) with four survival types, i.e. overall survival, disease-specific survival, disease-free interval, and progression-free interval, in 33 cancer types. Notably, the implemented AFT model provides an alternative method for genes/features which failed the proportional hazard assumption in Cox regression. With the unprecedented number of survival models implemented and high flexibility in analysis, DoSurvive is a unique platform for the identification of clinically relevant targets for cancer researcher and practitioners. DoSurvive is freely available at http://dosurvive.lab.nycu.edu.tw/.

## Highlights

• GUI platform for multivariate survival analysis with Cox regression or AFT
• Explore the prognostic power of single/multiple genes in cancers
• Investigate the prognostic power of methylation sites in cancers
• Access DoSurvive at http://dosurvive.lab.nycu.edu.tw/

## Graphical abstract

**Figure:**
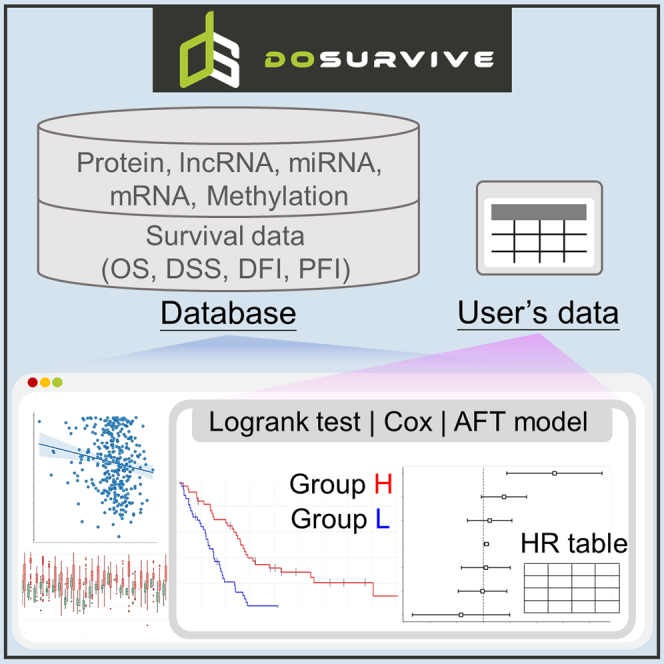

## Introduction

With the rise of precision medicine in cancer therapy, identification of biomarkers has become an important research issue.^1^ Biomarkers for diagnosis, prognosis or prediction of treatment response may be used for personalized therapy.^1^^,^^2^^,^^3^ Various online tools and databases have been developed to facilitate the discovery of potential prognosis biomarkers.^4^^,^^5^^,^^6^^,^^7^^,^^8^^,^^9^^,^^10^^,^^11^^,^^12^^,^^13^ For example, OncoLnc^4^ provides hazard ratios derived from Cox regression for expression levels of mRNA, miRNA or lncRNA of cancer tissues. Kaplan-Meier Plotter^8^ performs Log rank test of expression levels of mRNA, miRNA or lncRNA, and generates Kaplan-Meier plot for users to examine their possible correlations with cancer prognosis. Other versatile databases, including our previous work, TACCO,^5^ provide cancer survival analysis, detection of differentially expressed genes in cancer tissues, as well as correlation analysis for epigenetic regulations.^5^^,^^6^^,^^7^^,^^9^^,^^10^^,^^14^ These databases/webtools significantly facilitate the progression of cancer research, and hence are extensively used and frequently cited by the scientific communities.

Most of the existing databases provide implementation of Log rank test and Cox regression, which are the most well-known survival analysis methods. Log rank test is a nonparametric test used for comparison of survival data between two or more groups of individuals.^15^ Usually, patients are classified into two groups based on the expression values of the gene of interest, and then their survival data were compared with Log rank test. Cox regression can incorporate multiple explanatory variables including the gene expression levels and clinical characteristics. However, Cox regression makes the proportional hazards (PH) assumption on each variable in the model, while in practice not all features of interest satisfy the assumption.^16^^,^^17^ Recent studies have shown that many of the transcriptomic data in pan-cancer cohorts are in discrepancy with the PH assumption.^18^^,^^19^ Accelerated failure time (AFT) model is a useful alternative to Cox regression, even though it is relatively unfamiliar to most biologists. It directly models the effect of explanatory variables on the survival time instead of on the hazard function.^20^ AFT model usually provides approximately similar results as Cox regression.^21^^,^^22^ In addition, compared to the hazard ratios derived from Cox regression, the expected survival time derived from AFT model is more intuitive in terms of physiological interpretation.^22^

In addition to the statistical models, many of the existing prognosis biomarkers databases provide analysis only for single gene. The prognosis prediction power of single biomarker can be improved by combining with other biomarkers. Researchers have been searching for prognosis signatures, a set of biomarkers, of mRNA/miRNA/lncRNA/proteins in cancer tissue.^23^^,^^24^^,^^25^ However, among all the existing databases, only DriverDBv3 provides Log rank test results for predetermined gene pairs for users to explore potential synergistic effects.^10^ Given more and more studies showing combined effects of multiple genes in prognosis, and some proposed prognostic signatures are composed of miRNA, mRNA, and/or lncRNA, researchers are eager to explore the combined effects in cancer prognosis.^26^^,^^27^ A tool that allows customized selection of two or more biomarkers for prognostic analysis will be of great use.

Dysregulation of methylation at CpG islands in promoter regions may play important roles in the expression of downstream genes in cancer cells.^28^ Hence the methylation levels at CpG sites may also be potential biomarkers. However, the association between methylation at CpG sites and prognosis is barely addressed in the current existing cancer survival webtools.^29^

To tackle these unmet needs, we developed a database/webtool, DoSurvive, which is built-in with three survival statistical models including Log rank test, Cox regression, and AFT model. The webtool provides precalculated survival analysis for single mRNA, miRNA, lncRNA, protein and CpG methylation, and offers real time analyses for investigation into combined effects on prognosis for multiple miRNAs/mRNAs/lncRNAs and clinical factors simultaneously. Notably, DoSurvive is equipped with a user-friendly interface and allows users to upload their own dataset. Users also have the flexibility to download raw data and upload specific subsets of features or patients for reanalysis. The Kaplan-Meier plot generated by DoSurvive may be customized by users for publication purpose. DoSurvive is currently freely available for academic use at http://dosurvive.lab.nycu.edu.tw.

## Results and discussions

### Overview of DoSurvive

**Figure 1:**
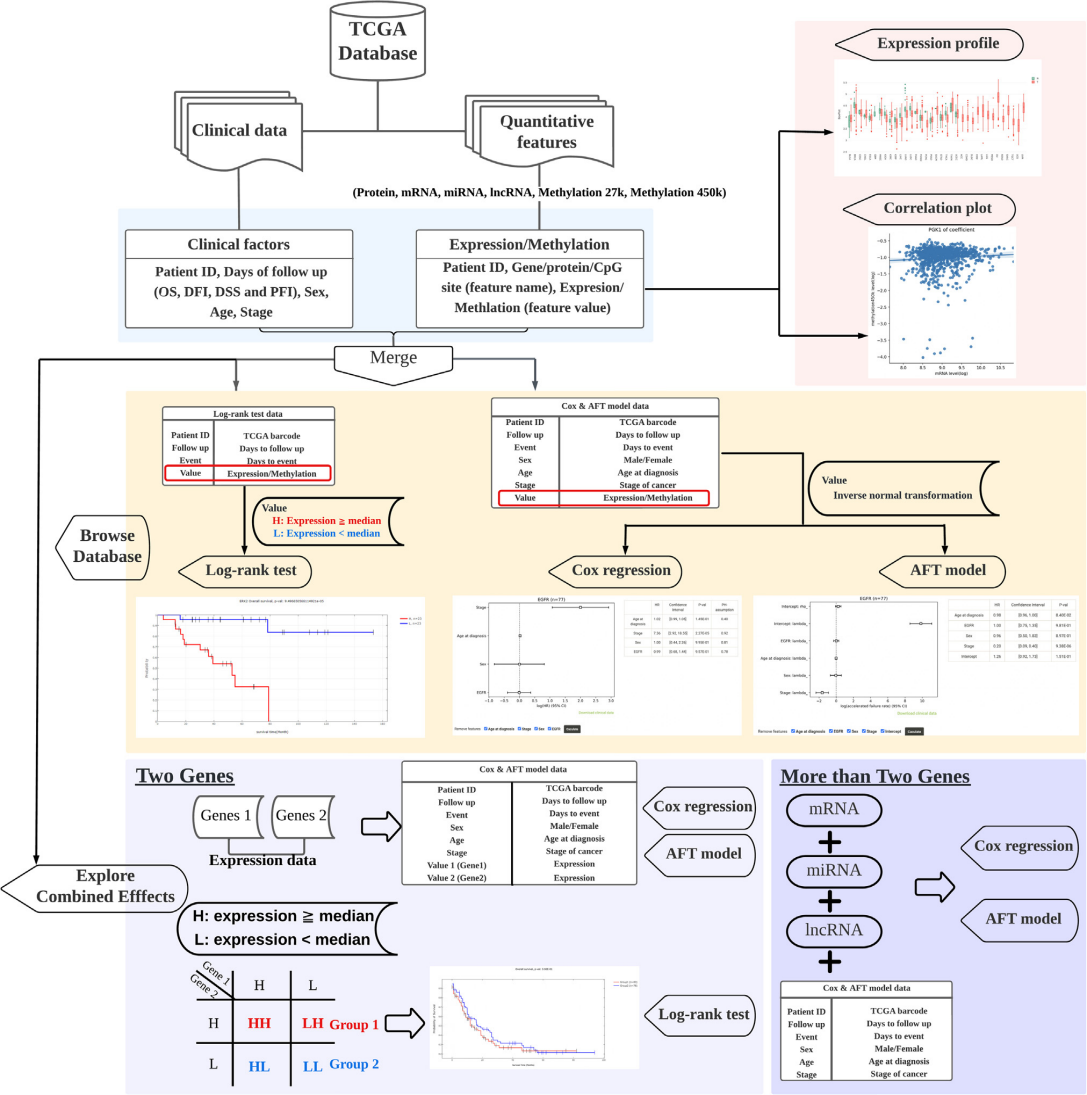
Construction of DoSurvive and output example All quantitative features and clinical data were downloaded from TCGA.^30^Users may browse the database (“Browse Database”; yellow section) or inquire into combined effects of multiple features (“Explore Combined Effects”; light and dark purple sections). In Browse Database section, users may exam the precalculated results from Log rank test, Cox regression or AFT model for single feature. In Explore Combined Effects section, users may opt for “Two Genes”, in which DoSurvive will perform Log rank test, Cox regression and AFT model analysis of two genes of user’s choice on the fly (light purple panel). Users may also opt for “More than Two Genes”, in which Cox regression and AFT model analysis will be performed of three or more genes of user’s choice (dark purple panel). OS: overall survival; DFI: disease-free interval; DSS: disease-specific survival; PFI: progression-free interval.

A summary of DoSurvive is shown in Figure 1. Three statistical survival analysis methods, Log rank test, Cox regression, and AFT model were implemented. DoSurvive supports survival analysis for a customized gene list, in which an interactive Kaplan-Meier plot and forest plots for the hazard ratio or time ratio may be generated. Additionally, users can upload their own dataset for survival analysis or download the raw data used in DoSurvive and customize the features or patients list included in Cox regression or AFT model analysis. DoSurvive offers the survival analysis for all the available quantitative features in TCGA,^30^ including expression levels of proteins, mRNAs, miRNAs, lncRNAs, and methylation levels at CpG sites. Users can browse the prognostic power of a single feature or inquire into the combined effects of multiple features in prognosis prediction in the four survival types (overall survival, disease-specific survival, disease-free interval, and progression-free interval). In addition to the survival analysis, DoSurvive also provides the expression profiles for all features across all cancer types and distribution plots for investigating possible correlation between methylation levels at CpG sites and mRNA levels of the downstream genes. All the analysis results, raw data used in statistical analysis or resulted plots in DoSurvive can be downloaded for further analyses.

DoSurvive links survival data to the levels of mRNA, miRNA, lncRNA, protein and methylation at CpG sites in 33, 33, 20, 32, and 33 cancer types, respectively. These quantitative features were analyzed with the four types of survival (*i.e.* overall survival, disease-free interval, disease-specific survival, and progression-free interval), separately. For each type of the endpoint, dozens to hundreds of patients were included in different cancer types for each feature (Figure S1). For a specific mRNA, miRNA, lncRNA, protein or methylation levels in a specific survival type, DoSurvive offers statistical results of survival derived from Log rank test, Cox regression model, and/or AFT model (Tables S1–S4).

### Browse database section

**Figure 2:**
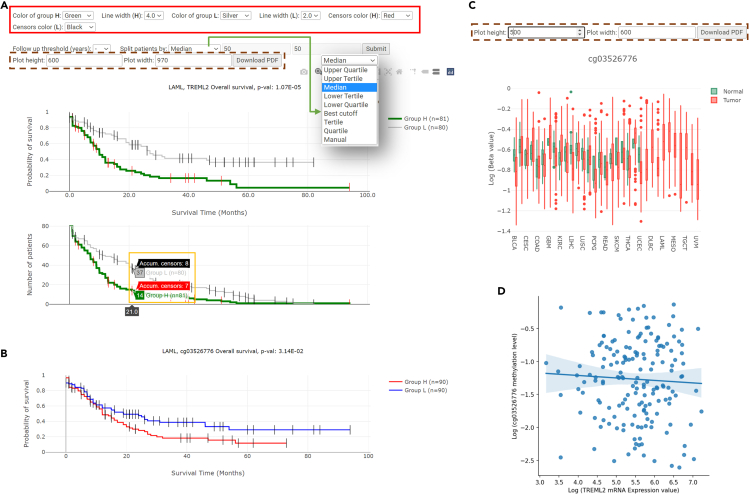
Kaplan-Meier plots for TREML2 and CpG site cg03526776, and their correlation in LAML (A) A customized Kaplan-Meier plot for overall survival. A total of 161 acute myeloid leukemia patients were grouped into Group H (high) and Group L (low) based on their expression levels of TREML2 mRNA. Group H has significantly worse survival. Group H and L were colored in green and silver, respectively. The red box highlights the visualization customization options available in DoSurvive. Users can adjust parameters such as line thickness and color. Additionally, they have the flexibility to modify the follow-up threshold or the cutoff to divide patients into two groups. As indicated by the green arrow, the cutoff can be manually defined or selected from suggested options such as the upper quartile, upper tertile, median, etc. The brown dashed-box indicates that users can resize the resulting Kaplan-Meier plot and download it as a PDF file. By hovering the mouse over the lower plot, users can obtain information about the number of patients at risk and the cumulative count of censored events at specific time points (yellow box). (B) Log rank test result for cg03526776 showing that significantly better overall survival in H group. (C) Methylation levels of cg03526776 across different cancer types. They green and red box represent methylation levels detected in normal and tumor tissues, respectively. Users can resize the boxplot and download it as a PDF file (as indicated by brown dashed-box). (D) The slightly negative correlation between expression level of TREML2 and methylation level of cg03526776.

For all the applicable features in every cancer type analyzed, DoSurvive contains the survival analysis results for the four types of endpoints using the three statistical methods. Users may browse the results for a specific gene, a specific cancer type, or a specific gene in a specific cancer type. For example, to infer potential prognostic mRNA biomarkers for overall survival in acute myeloid leukemia (LAML), one can click “Select a Cancer”, select “mRNA”, and choose “Acute Myeloid Leukemia (LAML)” from the dropdown list, and to obtain the survival analysis from Log rank test, Cox regression, and AFT model. Four significant genes may be found in Cox regression analysis: High expression levels of TREML2, ATP13A2, and MYH15 are significantly associated with shorter overall survival, and high expression level of C2orf67 (KANSL1L) is significantly associated with longer overall survival. These results are consistent with previous studies.^31^^,^^32^ TREML2 is also found to be significantly associated with overall survival in both Log rank test and AFT model. One may explore and customize the interactive Kaplan-Meier plot for TREML2. In addition to Kaplan-Meier plot, DoSurvive also provides information on the number of patients at risk and the cumulative count of censored events at various time points (Figure 2A).

DoSurvive also provides precalculated survival analysis for methylation levels at CpG sites around the gene of interest. Consistent with the general principle that higher CpG methylation level in the promoter regions correlates with lower gene expression, Kaplan-Meier plot showed higher methylation level of a CpG site, (cg03526776), near TREML2, associated with significantly better overall survival (Figure 2B). For users interested in exploring the biomarker potentials of CpG sites, DoSurvive also offers the distribution of the methylation levels across cancer types and the correlation between the expression level of genes of interest and the methylation levels of nearby CpG sites. For example, one may explore the methylation levels of cg03526776 in tumor tissues and adjacent normal tissues, if available, across different cancer types (Figure 2C). The distribution plot shows slightly negative correlation between the expression levels of TREML2 and methylation levels of cg03526776 in LAML patients (Figure 2D).

**Figure 3:**
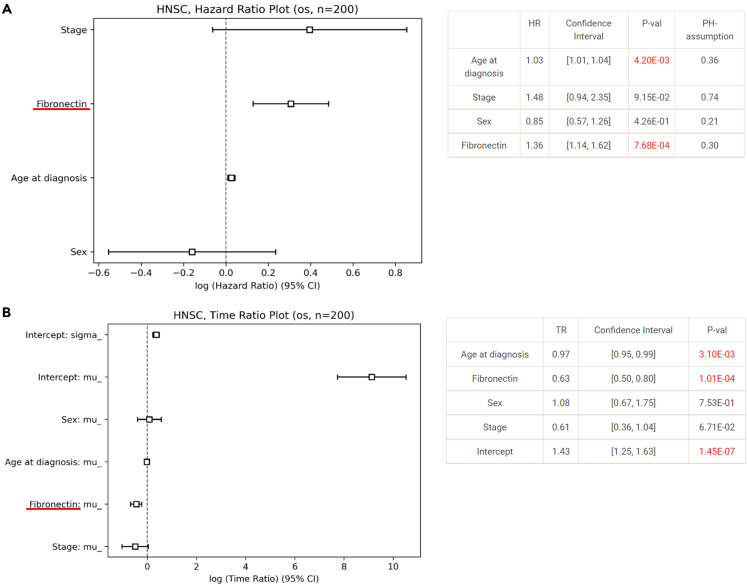
Analysis of association between overall survival and protein fibronectin in 200 HNSC patients using Cox regression and AFT model (A) Hazard ratios calculated with Cox regression analysis for all the tested variables. DoSurvive presents the hazard ratios and their confidence intervals in a forest plot and a table. DoSurvive also checks the PH assumption. In forest plot, the hazard ratios of the features and clinical characteristics are plotted together with their confidence intervals. The variables on the *y* axis are ordered based on their hazard ratios. Both age at diagnosis and fibronectin levels have hazard ratios significantly larger than one and therefore positive log values of the hazard ratios. (B) A forest plot and a table of the time ratios and confidence intervals calculated from AFT model. Both age at diagnosis and fibronectin levels have time ratios significantly smaller than one and therefore negative log values of the time ratios.

The protein expression level of fibronectin is considered a potential biomarker in head and neck cancer (HNSC).^33^^,^^34^ Consistently, fibronectin was also found to be significant by both Cox regression and AFT model with p values of 7.68 × 10^−4^ and 1.01 × 10^−4^, respectively. The forest plots and tables of hazard ratios or time intervals derived from Cox regression and AFT models for fibronectin, age, sex, and cancer stage are shown in Figure 3. The protein level of fibronectin and age at diagnosis are both found significantly associated with overall survival in both Cox regression and AFT models. In Cox regression, fibronectin has a coefficient significantly larger than 0 and the confidence interval is between 1.14 and 1.62, suggesting that fibronectin is a risk factor. Consistently, fibronectin is also found to be a significant risk factor in AFT model. In AFT model, fibronectin has a negative coefficient, which is correlated with shorter survival time.^35^ The confidence interval for fibronectin is between 0.50 and 0.80.

**Figure 4:**
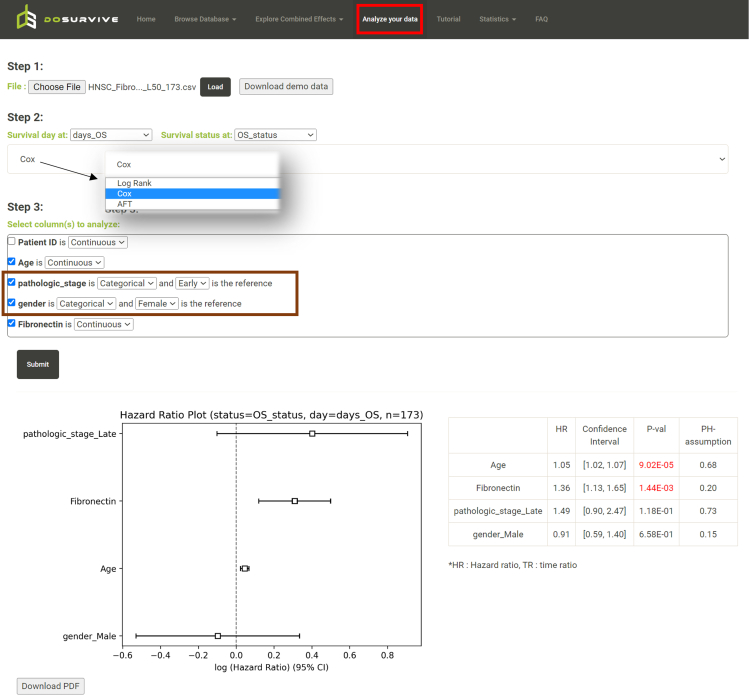
Reanalysis of the association between overall survival and fibronectin in older HNSC patients using Cox regression The Cox regression analysis was performed on a cohort of 173 HNSC patients aged 50 years and above, selected from the original pool of 200 HNSC patients presented in Figure 3A. The results indicate that both age and fibronectin levels are identified as significant risk factors, with hazard ratios significantly higher than one (p value = 9.02E-05 and 1.44E-3, respectively). In the “Analyze your data” function (indicated by the red box), users can upload a dataset for various statistical models such as the Log rank test, Cox regression, or AFT model. When selecting Cox regression or AFT model, users are prompted to choose the specific column(s) for analysis. For each chosen column, users must indicate whether it represents a Continuous or Categorical variable. In the case of categorical features, users also need to select the reference category (brown box).

DoSurvive also allows users to analyze their own dataset. For example, if one is interested in the expression of fibronectin in prognosis of older patients of HNSC, one may download the clinical data and remove the 27 patients who are less than 50 years old. As shown in Figure 4, after uploading the updated file and reanalyzing with Cox regression with “Analyze your data”, one may find fibronectin still being a significant risk factor with a confidence interval between 1.13 and 1.65.

### Inquiry into combined effects of multiple genes in real time

**Figure 5:**
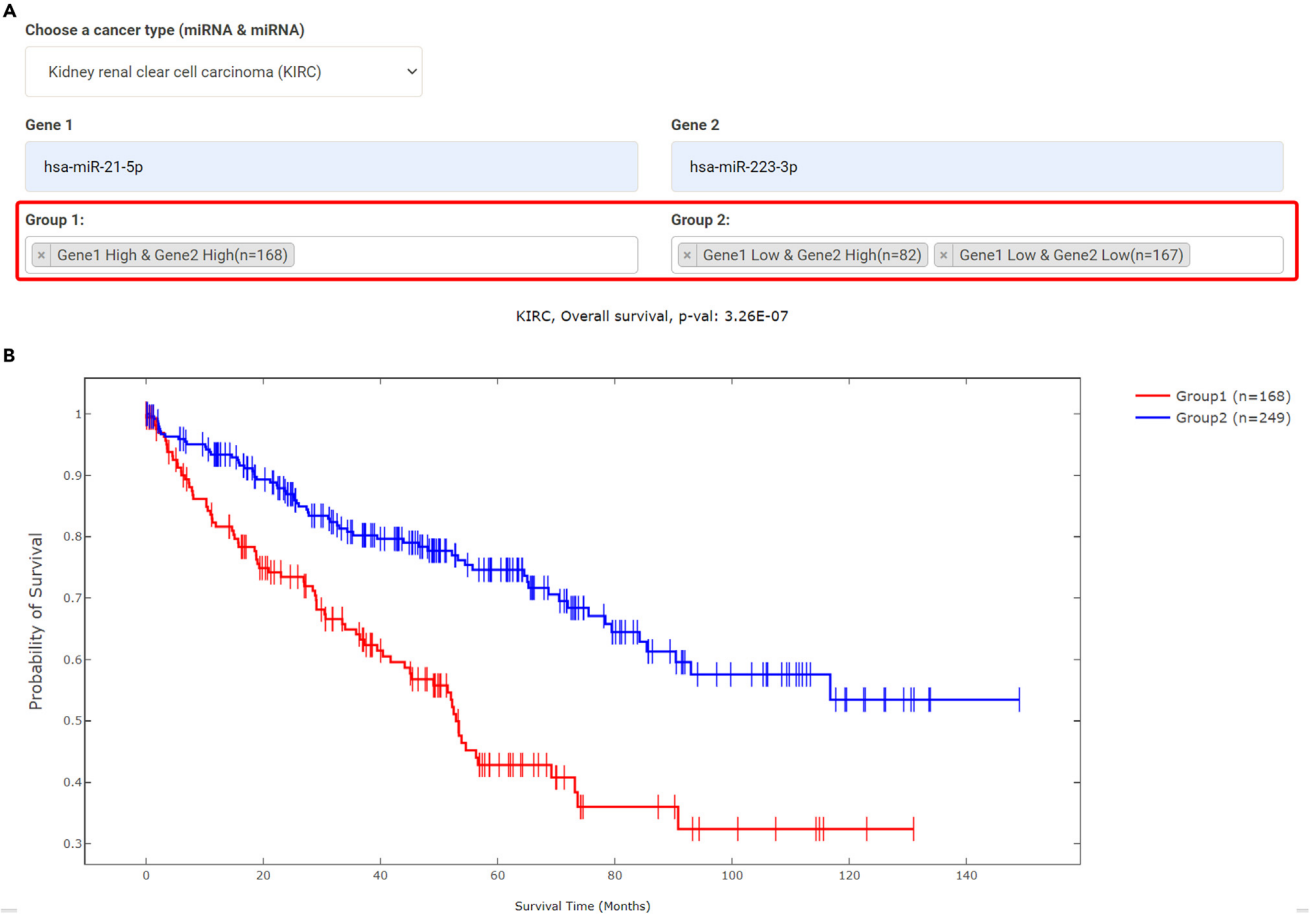
Example for Log rank test in Two Genes (A) Based on the median expression levels of two miRNAs (has-miR-21-5p and has-miR-223-3p), all the KIRC patients may be classified into four groups: (1) high in both miRNAs, (2) low in both miRNAs, (3) high in the first but low in the second, and (4) low in the first but high in the second. (B) Overall survival between patients in Group 1 and Group 2 are compared in DoSurvive with Log rank test and presented in an interactive Kaplan-Meier plot.

The expression level of multiple genes may be used together to predict prognosis of cancers. For example, hsa-miR-21-5p and hsa-miR-223-3p were reported as a signature for both metastasis and overall survival in kidney renal clear cell carcinoma (KIRC).^36^ In DoSurvive, one may choose “Explore Combined Effects,” “Two Genes,” “miRNA and miRNA,” “Choose a cancer type,” “KIRC,” and enter “hsa-miR-21-5p,” and “hsa-miR-223-3p.” DoSurvive groups KIRC patients into four groups based on the median expression levels of the two miRNAs and allows user to compare the survival among the patient groups with Log rank test (Figure 5). The four patient groups may be compared against one another in any combinations. The results confirmed that hsa-miR-21-5p and hsa-miR-223-3p significantly associated with both overall survival in Cox regression and AFT model. These two miRNAs were also found significantly associated with disease-specific survival in Cox regression (Table 1).

**Table 1:** Cox regression and AFT model results for hsa-miR-21-5p and hsa-miR-223-3p in KIRC from Two Genes analysis in DoSurvive

	Survival type	Number of patients	Coefficient (hsa-miR-21-5p)	P-val (hsa-miR-21-5p)	Coefficient (hsa-miR-223-3p)	P-val (hsa-miR-223-3p)
Cox regression	Overall	494	2.42E-01	1.22E-02^a^	2.34E-01	8.92E-03^a^
Cox regression	Disease-Free	107	−2.84E-01	3.73E-01	−3.04E-01	3.60E-01
Cox regression	Progression-Free	492	8.97E-02	3.57E-01	1.66E-01	6.90E-02
Cox regression	Disease-Specific	482	2.69E-01	2.61E-02^a^	2.49E-01	2.13E-02^a^
AFT model	Overall	494	−2.3E-01	3.9E-02^a^	−2.9E-01	8.0E-03^a^
AFT model	Disease-Free	107	4.9E-01	2.3E-01	2.3E-01	5.3E-01
AFT model	Progression-Free	492	−3.7E-02	7.7E-01	−2.4E-01	4.8E-02^a^
AFT model	Disease-Specific	482	−2.1E-01	8.3E-02	−3.1E-01	1.1E-02^a^

a Significant, i.e. p- value <0.05.

**Figure 6:**
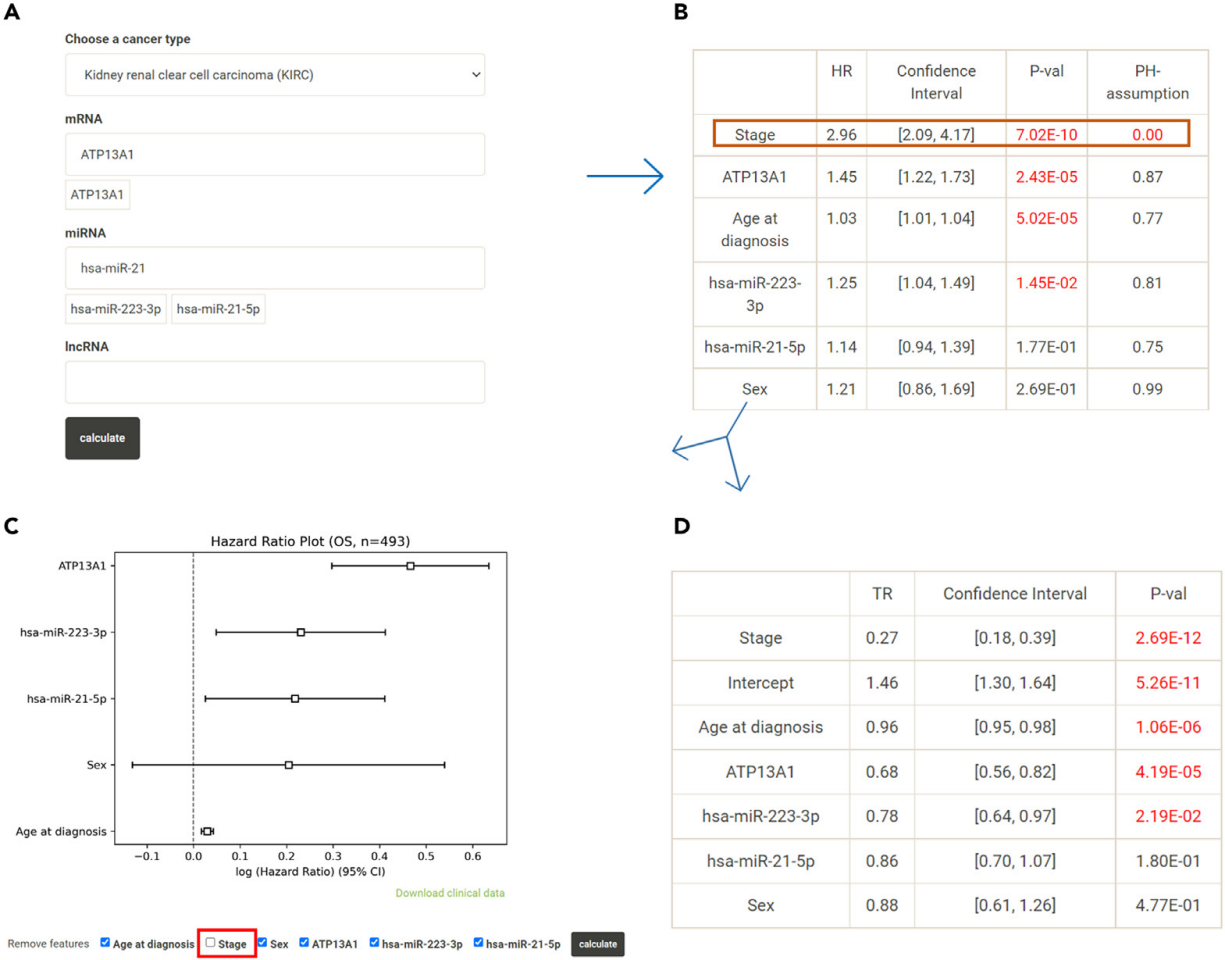
Example of “More than Two Genes” and solutions for failure in PH assumption (A) Cox regression and AFT model analysis may be performed in DoSurvive for selected genes and clinical factors. (B) This example shows “Stage” failing the PH assumption (brown box) and should be excluded in Cox regression model. DoSurvive provides two solutions to this failure. (C) First solution: Reperforming the Cox regression model without Stage (red box). (D) Second solution: Using AFT model.

To demonstrate the function of “More than Two Genes,” top KIRC overall survival-associated mRNAs in Log rank test, ATP13A1 found in DoSurvive was analyzed together with hsa-miR-21-5p and hsa-miR-223-3p. Both Cox regression and AFT model revealed that both ATP13A1 and hsa-miR-223-3p, Cancer Stage and patient’s age are significantly associate with overall survival in multivariant analysis. Notably, in Cox regression, Cancer Stage violates the PH assumption. To perform a valid survival analysis, one may choose to uncheck “Stage” and redo Cox regression analysis or use the survival analysis result from the alternative AFT model instead (Figure 6). Notably, in results from AFT model, Cancer Stage is found to be a significant risk factor, which could not be analyzed with Cox regression.

### AFT model contributes to the identification of survival-associated genes which violate the PH assumption

A distinguishing feature of DoSurvive is to provide the AFT model which is a popular alternative to the Cox regression model. Log rank test and Cox regression are the most well-known methods for identification of potential biomarkers related to patients’ survival. They both have their own limitations. Log rank test treats all patients in the test equally as belonging to the same group, even if they usually have different expression values for a specific gene. Also, relevant clinical characteristics can’t be included in Log rank test. Cox regression can incorporate clinical characteristics as well as the expression values in the analysis. However, the PH assumption may not hold for some explanatory variables. Notably, not all the cancer researchers check the PH assumption, when they use Cox regression in their studies. A review article shows that only 5% of the studies published in cancer journals have tested for the assumption.^37^ Similarly, a recent review of 115 non-small cell lung cancer studies found that only four (3%) had tested for the PH assumption.^38^ In contrast, a more recent review of articles in the field of total joint arthroplasty utilizing Cox regression analysis found that 19.8% of the studies had tested for the PH assumption.^39^ These findings suggest a need for increased attention to testing for the PH assumption in Cox regression analysis. Therefore, we provide the AFT model as an alternative solution in DoSurvive.

Compared to other existing webtools, the AFT model is a unique feature of DoSurvive, offering an alternative multivariant analysis method on features that fail PH assumption (Table 2). As shown in Figure 6, the factors that fail the PH assumption may nevertheless turn out to be significantly associated with survival. For example, in bladder urothelial carcinoma, TNFRSF14 which fails PH assumption, was found significantly associated with overall survival in both Log rank test and AFT model. Moreover, the coefficient for TNFRSF14 is positive in AFT model, implying that higher TNFRSF14 expression is associated with longer survival interval. Indeed, increased expression of TNFRSF14 was reported to promote apoptosis and suppress proliferation in bladder cancer.^40^

**Table 2:** Comparison of DoSurvive with other survival analysis webtools

	Oncolnc	UALCAN	KM-plotter	R2	DriverDBv3	TANRIC	cBioPortal	GEPIA	PRECOG	PROGgeneV2	DoSurvive
# of cancer types	**21**	**31**	**21**	**33**	**33**	**33**	**33**	**33**	**33**	**27**	**33**
Log Rank test	**+**	**+**	**+**	**+**	**+**	**+**	**+**	**+**	**+**	**+**	**+**
Kaplan-Meier plot	**+**	**+**	**+**	**+**	**+**	**+**	**+**	**+**	**+**	**+**	**+**
Cox regression	**+**		**+**		**+**	**+**		**+**	**+**	**+**	**+**
AFT model											**+**
Comparison of expression in N/T		**+**	**+**	**+**	**+**		**+**	**+**			**+**
Patient filtering		**+**	**+**	**+**	**+**	**+**	**+**				**+**
Survival analysis for CpG sites							**+**^b^				**+**
CpG site and mRNA correlation					**+**		**+**^b^				**+**
# of feature types	**3**	**2**	**3**	**3**	**4**	**3**	**2**	**3**	**3**	**2**	**5**
Survival analysis for multiple genes					**+**^a^						**+**
# of Survival types	**1**	**1**	**4**	**1**	**4**	**1**	**4**	**2**	**2**	**3**	**4**
Customization of Kaplan-Meier plot				**+**				**+**			**+**
Analysis of user’s dataset			**+**	**+**							**+**

a DriverDBv3 provides Hazard Ratio for predetermined gene pairs.

b For each gene, cBioPortal provides survival analysis and correlation only for the upstream CpG site that show highest correlation with that selected gene.

### Advantages of DoSurvive

DoSurvive incorporates widely used survival analysis methods to investigate the relationship between survival and independent categorical/continuous covariates. The Log rank test is widely utilized to assess disparities in survival between different groups. As a non-parametric method, the Log rank test does not rely on any assumptions regarding the data distributions. On the other hand, Cox regression is a semi-parametric approach frequently used to investigate the relationship between survival and multiple features. However, Cox regression requires the proportional hazard (PH) assumption, which has been found to be violated in many transcriptome datasets.^18^^,^^19^ The AFT model, which describes the relationship between the logarithm of the survival time and the covariates, is a popular alternative commonly applied to datasets that violate the PH assumption.^21^^,^^22^

### Implementation of DOSurvive

The development of DoSurvive involved the integration of Django,^47^ JavaScript, and MongoDB.^48^ Single variant survival analysis was pre-computed using Python, and the results were stored in MongoDB, a NoSQL database, categorized by different cancer types. The multivariant survival analysis is performed in real-time upon user feature selection. To ensure a seamless user experience, we designed a user-friendly web interface using HTML, CSS, and JavaScript for the frontend. Data retrieval from MongoDB is facilitated by Django. This interface empowers users to effortlessly execute queries and access the stored data, enhancing the tool’s usability and efficiency.

### Limitations of the study

The number of cancer patients in the TCGA dataset varies across different cancer types (Figure S1), and in some cancer types, the number of events (death or disease progression) is limited. A general guideline recommends the events per variable guideline, suggesting a minimum of ten events per predictor variable.^41^^,^^42^ However, in certain cases, this rule of thumb can be relaxed.^43^ Nevertheless, it is generally advisable to prioritize a larger sample size and an increased number of events to achieve higher accuracy in survival analysis. Therefore, it is crucial for users to be aware that the predictive accuracy of DoSurvive may be compromised in certain cancer types due to the limitations imposed by the sample size and the censoring rate. Users should also investigate the length of follow-up to understand the reasons for the phenomenon of few events compared with the sample size. If it is determined that the scarcity of events is indeed due to their rarity, users should explore alternative methods that are specifically designed to analyze and address research interests pertaining to rare events.^44^

In order to accommodate a wide user base, DoSurvive offers these three methods, which encompass a significant portion of survival analysis across multiple cancer types. However, we acknowledge that certain limitations exist. The survival analysis performed in DoSurvive assumes a single type of outcome, where patients are classified as either 0 (censored) or 1 (event occurred). In some cases, researchers may be interested in jointly analyzing multiple types of outcomes, such as relapse and death. For such projects, utilizing multi-state models may be a more suitable option to adequately address the research interest.^45^ Additionally, when studying longitudinal covariates such as the progression of circulating tumor cells over time, joint models that simultaneously consider both the longitudinal measurements and survival processes are a useful statistical tool for analyzing and interpreting this type of longitudinal data.^46^ In these specific cases, alternative statistical methods are required to ensure a comprehensive analysis of survival data.

## STAR★Methods

### Key resources table

**Table:** 

REAGENT or RESOURCE	SOURCE	IDENTIFIER
**Software and algorithms**		

Mongo/4.0.3	mongo	https://www.mongodb.com/
Python/3.9.16	Python Software Foundation	https://www.python.org/
Django/4.1.7	Django Software Foundation	https://www.djangoproject.com/
djangorestframework/3.14.0	djangorestframework	https://www.django-rest-framework.org/
lifelines/0.27.4	lifelines	https://github.com/CamDavidsonPilon/lifelines
matplotlib/3.7.0	matplotlib	https://matplotlib.org/
numpy/1.24.2	numpy	https://www.numpy.org/
pandas/1.5.3	pandas	https://pandas.pydata.org/
plotly/5.13.1	plotly	https://plotly.com/python/
pymongo/4.3.3	The MongoDB Python Team	http://github.com/mongodb/mongo-python-driver
scipy/1.10.1	scipy	https://scipy.org/
seaborn/0.12.2	seaborn	https://github.com/mwaskom/seaborn

### Resource availability

#### Lead contact

Further information and any related requests should be directed to and will be fulfilled by the lead contact, Prof. Ting-Wen Chen (dodochen@nctu.edu.tw).

#### Materials availability

This study did not generate new unique reagents.

### Method details

#### Data acquisition

The expression data for lncRNA were downloaded from TANRIC database.^9^ The batch corrected expression levels of mRNA, miRNA and protein, clinical data, and survival information for all the cancer patients were downloaded from Pan-Cancer Atlas website (https://gdc.cancer.gov/about-data/publications/pancanatlas).^30^ The methylation data for 33 cancer types were downloaded from Broad GDAC Firehose with firehose get and the downloaded version was released on 2016/01/28 (http://firebrowse.org/).

#### Database and website implementation

All the analyzed results and raw data were stored in MongoDB database.^48^ Python web framework, Django^47^ was used to connect to the database and to build the website. When users click the button of analysis, DoSurvive retrieves the selected raw data from database, executes the analysis and generates the interactive Kaplan-Meier plot, box plot or forest plot on the fly. JavaScript, CSS, inhouse Python scripts and HTML were used in front-end website development.

### Quantification and statistical analysis

The quantitative features, *i.e.*, expression values of mRNA, miRNA, lncRNA and proteins, and the methylation levels at CpG sites, are used in the survival analysis. To minimize potential noise, proteins detected in more than 50% of the samples and other features detected in more than 75% of the samples with the median value larger than cutoffs were used for statistic tests. The cutoffs for mRNA, lncRNA, and methylation sites are 1.00, 0.02, and 0.02, respectively. For each cancer type, all the qualified features were tested with all three survival analysis methods, Log-rank test, Cox regression and the AFT model with the Python package, “lifelines”.^49^

To perform a Log-rank test, all patients of a chosen cancer type were divided into two groups at the median of the tested feature and the survival time of the two groups were compared. Cox regression and the AFT model incorporate more explanatory variables such as a patient’s sex, age and cancer stage when applicable. Specifically, a patient’s sex was treated as a binary variable and age was treated as a continuous variable. The cancer stage was further grouped into early (stage I and stage II) and late (stage III/stage IV) stages and treated as a binary variable. The cancer stage was classified based on patient’s pathological stage or the histological stage if the pathological stage is not available. To avoid the bias caused by extreme outliers, the expression levels and the methylation levels were normalized by rank-based inverse normal transformation.^4^^,^^50^ With PH assumption being required for Cox regression, only features satisfying this assumption were included in the Cox regression.

Four types of time-to-event endpoints are provided: (1) overall survival, (2) disease-specific survival, (3) disease-free interval, and (4) progression-free interval. For each survival analysis, only patients having complete required clinical information were included in the analyses. Not all the patients from Pan-Cancer Atlas^30^ had all the four types of endpoints, the numbers of tested patients vary across the different survival types of endpoints for the same cancer type. DoSurvive presents the tested results as well as the number of patients used in the analysis. Given multiple tests were performed for each survival estimation, Benjamini & Hochberg multiple test correction was applied to the *p*-values from survival analysis for each feature in each cancer type.^51^

## Data Availability

• This paper does not generate original data. The sources of the pubilc datasets used are presented in the materials availability section. • Source code for DoSurvive is freely available for download at https://github.com/dodolab435/python_nctu_cancer. • Any additional information is available from the lead contact upon request.
