# Supplementary material for: DoSurvive: A webtool for investigating the prognostic power of a single or combined cancer biomarker
Source: iScience. 2023 Jul 4;26(8):107269. doi: 10.1016/j.isci.2023.107269 (PMC10440714; doi:10.1016/j.isci.2023.107269)
Supplement: Document S1. Figure S1 and Tables S1–S4 [file mmc1.pdf]

iScience, Volume *26*

Supplemental information

DoSurvive: A webtool for investigating the prognostic power of a single or combined cancer biomarker

Hao-Wei Wu, Jian-De Wu, Yen-Ping Yeh, Timothy H. Wu, Chi-Hong Chao, Weijing Wang, and Ting-Wen Chen

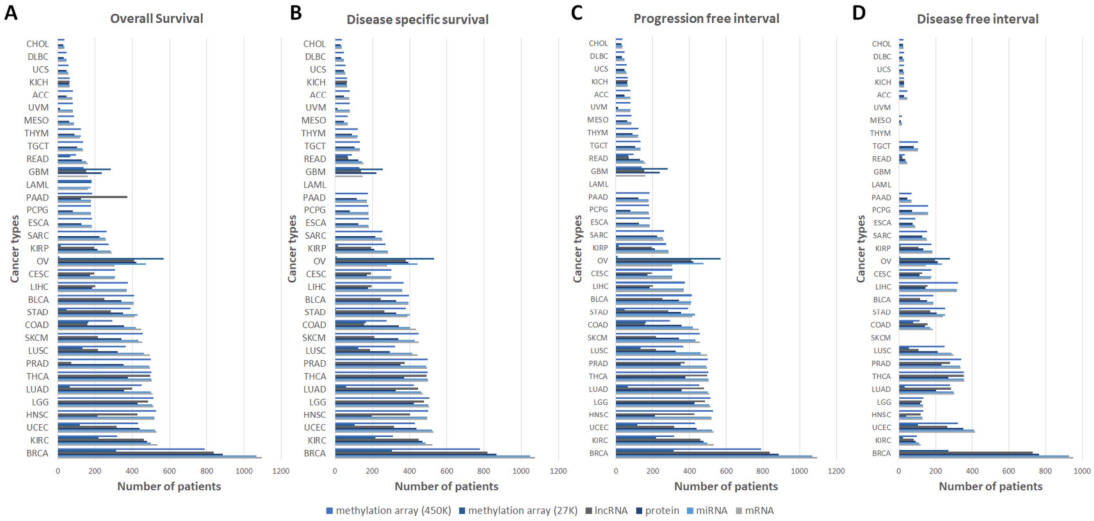

Figure S1. Bar plots for the number of patients tested with log-rank test in DoSurvive across different cancer types, related to Figure 1. (A) A bar plot shows the number of patients tested in different feature types for overall survival in 33 cancer types. (B) A bar plot shows the number of patients tested for disease specific survival (DSS). There is no DSS information for LAML. (C) A bar plot shows the number of patients tested for progression free interval (PFI). No PFI information is available for LAML. (D) Number of patients tested for disease free interval (DFI). There is no DFI information for LAML, GBM, SKCM, THYM and UVM.

Supplementary Table 1. Number of features tested in DoSurvive (Overall survival), related to Figure 1.

	mRNA			miRNA			lncRNA			Protein		
	Log-rank	Cox	AFT	Log-rank	Cox	AFT	Log-rank	Cox	AFT	Log-rank	Cox	AFT
**ACC**	15,849	14,708	15,865	523	44,684	523	-	-	-	194	176	194
**BLCA**	16,381	14,061	16,381	532	394	532	4,056	3,798	4,053	196	190	196
**BRCA**	16,665	13,158	16,674	492	450	492	4,537	4,045	4,538	195	150	195
**CESC**	16,362	15,029	16,367	534	467	534	4,128	3,906	4,125	193	181	193
**CHOL**	16,379	16,168	16,379	518	511	518	0	0	0	194	192	194
**COAD**	16,374	14,653	16,373	574	528	578	1,216	1,164	1,213	196	189	196
**DLBC**	15,843	14,838	15,796	495	471	502	-	-	-	194	176	194
**ESCA**	16,716	15,695	16,728	515	478	516	-	-	-	193	188	193
**GBM**	16,829	14,825	16,829	-	-	-	4,940	4,638	4,940	191	183	191
**HNSC**	16,653	16,213	16,648	541	536	543	3,829	3,488	3,804	191	177	191
**KICH**	16,392	15,102	16,396	481	460	481	4,090	3,717	4,085	190	185	190
**KIRC**	16,692	15,353	16,693	460	421	461	5,012	4,670	5,009	196	186	196
**KIRP**	16,478	15,641	16,484	480	480	482	4,351	4,082	4,339	197	192	194
**LAML**	15,136	13,415	15,136	396	381	392	-	-	-	-	-	-
**LGG**	16,803	13,963	16,807	553	376	553	5,316	4,550	5,316	193	158	193
**LIHC**	15,855	13,856	15,845	517	472	513	3,395	3,075	3,386	190	157	190
**LUAD**	16,790	15,718	16,797	519	493	518	4,556	4,368	4,573	196	192	196
**LUSC**	16,951	16,293	16,956	525	493	525	4,619	4,355	4,625	196	189	196
**MESO**	16,566	15,719	16,568	531	505	531	-	-	-	190	179	190
**OV**	16,805	15,392	16,803	459	388	459	4,871	4,533	4,863	196	181	196
**PAAD**	17,208	15,886	17,210	538	508	539	-	-	-	197	186	197
**PCPG**	16,409	15,242	16,409	529	475	529	-	-	-	193	186	185
**PRAD**	16,802	15,578	16,802	477	445	477	4,566	4,323	4,566	193	185	193
**READ**	16,464	14,494	16,465	570	509	567	1,242	1,188	1,244	191	186	191
**SARC**	16,219	14,773	16,219	481	403	481	-	-	-	193	179	193
**SKCM**	16,052	15,300	16,054	532	515	530	3,961	3,847	3,977	196	184	196
**STAD**	16,800	15,780	16,743	507	501	510	4,815	4,569	4,806	193	182	193
**TGCT**	17,466	17,480	15,944	670	676	638	-	-	-	194	194	108
**THCA**	16,464	15,941	16,468	513	507	513	4,495	4,393	4,496	194	192	194
**THYM**	16,626	16,360	16,612	615	587	613	-	-	-	194	189	194
**UCEC**	16,659	15,518	16,661	520	498	519	1,335	1,220	1,335	196	189	196
**UCS**	16,947	16,331	16,968	562	534	562	-	-	-	193	168	193
**UVM**	15,584	13,624	15,544	496	448	489	-	-	-	193	193	149

Supplementary Table 2. Number of features tested in DoSurvive (Progression free interval), related to Figure 1.

	mRNA			miRNA			lncRNA			Protein		
	Log-rank	Cox	AFT	Log-rank	Cox	AFT	Log-rank	Cox	AFT	Log-rank	Cox	AFT
**ACC**	15,849	14,877	15,865	523	486	523	-	-	-	194	173	194
**BLCA**	16,381	14,461	16,380	532	473	532	4,056	3,775	4,052	196	168	196
**BRCA**	16,665	12,635	16,674	492	444	492	4,537	3,958	4,538	195	172	195
**CESC**	16,362	15,149	16,367	534	491	534	4,128	3,845	4,125	193	165	193
**CHOL**	16,379	14,418	16,379	518	473	518	-	-	-	194	192	194
**COAD**	16,374	14,917	16,375	574	518	575	1,216	1,145	1,213	196	185	196
**DLBC**	15,843	15,108	15,796	495	482	502	-	-	-	194	186	194
**ESCA**	16,716	14,366	16,728	515	452	516	-	-	-	193	188	193
**GBM**	16,829	15,053	16,829	-	-	-	4,940	4,640	4,940	191	184	191
**HNSC**	16,653	15,648	16,648	541	518	543	3,829	3,414	3,804	191	179	191
**KICH**	16,392	15,573	16,396	481	457	481	4,090	3,844	4,085	190	167	190
**KIRC**	16,692	15,975	16,686	460	447	462	5,012	4,410	5,009	196	189	196
**KIRP**	16,478	15,833	16,488	480	462	482	4,351	4,102	4,345	197	181	194
**LAML**	-	-	-	-	-	-	-	-	-	-	-	-
**LGG**	16,803	14,678	16,807	553	474	553	5,316	4,810	5,316	193	185	193
**LIHC**	15,855	13,278	15,839	517	438	512	3,395	3,218	3,396	190	186	190
**LUAD**	16,790	15,703	16,797	519	496	518	4,556	4,329	4,573	196	179	196
**LUSC**	16,951	16,119	16,956	525	494	525	4,619	4,268	4,625	196	186	196
**MESO**	16,566	15,995	16,568	531	503	531	-	-	-	190	183	190
**OV**	16,805	15,768	16,803	459	426	459	4,871	4,461	4,863	196	177	196
**PAAD**	17,208	12,903	17,210	538	465	539	-	-	-	197	182	197
**PCPG**	16,409	15,402	16,412	529	518	529	-	-	-	193	191	193
**PRAD**	16,802	16,141	16,802	477	445	477	4,566	4,323	4,566	193	185	193
**READ**	16,464	14,669	16,463	570	509	567	1,242	1,188	1,244	191	186	191
**SARC**	16,219	14,290	16,219	481	403	481	-	-	-	193	179	193
**SKCM**	16,052	14,775	16,053	532	515	530	3,961	3,847	3,977	196	184	196
**STAD**	16,800	15,775	16,742	507	501	510	4,815	4,569	4,806	193	182	193
**TGCT**	17,466	15,421	17,480	670	676	638	-	-	-	194	194	108
**THCA**	16,464	15,995	16,468	513	507	513	4,495	4,393	4,496	194	192	194
**THYM**	16,626	15,312	16,612	615	587	613	-	-	-	194	189	194
**UCEC**	16,659	15,640	16,661	520	498	519	1,335	1,220	1,335	196	189	196
**UCS**	16,947	16,193	16,968	562	534	562	-	-	-	193	168	193
**UVM**	15,584	14,943	15,546	496	448	489	-	-	-	193	193	149

Supplementary Table 3. Number of features tested in DoSurvive (Disease free interval), related to Figure 1.

	mRNA			miRNA			lncRNA			Protein		
	Log-rank	Cox	AFT	Log-rank	Cox	AFT	Log-rank	Cox	AFT	Log-rank	Cox	AFT
**ACC**	15,849	13,670	15,818	523	488	524	-	-	-	194	186	194
**BLCA**	16,381	15,818	16,343	532	515	526	4,056	3,828	4,038	196	186	196
**BRCA**	16,665	15,429	16,663	492	469	491	4,537	4,320	4,524	195	180	195
**CESC**	16,362	15,628	16,345	534	521	538	4,128	3,980	4,112	193	158	193
**CHOL**	16,379	15,137	16,332	518	483	518	-	-	-	194	183	194
**COAD**	16,374	16,037	16,303	574	532	535	1,216	1,143	1,178	196	187	196
**DLBC**	15,843	15,751	-	495	506	-	-	-	-	194	-	-
**ESCA**	16,716	14,842	16,710	515	484	520	-	-	-	193	190	193
**GBM**	-	-	-	-	-	-	-	-	-	-	-	-
**HNSC**	16,653	14,613	16,597	541	475	533	3,829	3,462	3,780	191	189	191
**KICH**	16,392	15,053	14,978	481	454	448	4,090	3,543	3,759	190	169	178
**KIRC**	16,692	16,490	16,569	460	456	458	5,012	4,543	4,677	196	189	195
**KIRP**	16,478	15,775	16,410	480	456	477	4,351	3,812	4,319	197	167	194
**LAML**	-	-	-	-	-	-	-	-	-	-	-	-
**LGG**	16,803	16,197	16,781	553	548	558	5,316	5,023	5,263	193	165	193
**LIHC**	15,855	13,997	15,805	517	459	514	3,395	3,283	3,380	190	188	190
**LUAD**	16,790	16,138	16,847	519	510	519	4,556	4,405	4,668	196	177	196
**LUSC**	16,951	16,111	16,967	525	498	530	4,619	4,394	4,642	196	168	196
**MESO**	16,566	-	-	531	-	-	-	-	-	190	-	-
**OV**	16,805	16,127	16,786	459	431	456	4,871	4,368	4,865	196	182	192
**PAAD**	17,208	15,666	17,253	538	499	546	-	-	-	197	180	196
**PCPG**	16,409	13,103	16,433	529	469	532	-	-	-	193	193	127
**PRAD**	16,802	15,412	16,797	477	455	478	4,566	4,389	4,583	193	188	193
**READ**	16,464	16,471	16,533	570	583	583	1,242	1,262	380	191	177	191
**SARC**	16,219	13,509	16,216	481	413	472	-	-	-	193	174	193
**SKCM**	-	-	-	-	-	-	-	-	-	-	-	-
**STAD**	16,784	15,414	16,666	507	487	501	4,815	4,696	4,923	193	189	193
**TGCT**	17,466	13,753	17,535	670	562	673	-	-	-	194	190	194
**THCA**	16,464	15,896	16,464	513	464	514	4,495	4,231	4,513	194	177	194
**THYM**	-	-	-	-	-	-	-	-	-	-	-	-
**UCEC**	16,659	15,930	16,646	520	503	520	1,335	1,256	1,337	196	192	196
**UCS**	16,947	16,467	16,902	562	547	561	-	-	-	193	188	193
**UVM**	-	-	-	-	-	-	-	-	-	-	-	-

Supplementary Table 4. Number of features tested in DoSurvive (Disease specific survival), related to Figure 1.

	mRNA			miRNA			lncRNA			Protein		
	Log-rank	Cox	AFT	Log-rank	Cox	AFT	Log-rank	Cox	AFT	Log-rank	Cox	AFT
**ACC**	15,849	14,666	15,878	523	502	523	-	-	-	194	176	194
**BLCA**	16,381	13,097	16,385	532	377	532	4,056	3,656	4,043	196	169	196
**BRCA**	16,665	12,723	16,673	492	430	493	4,537	3,788	4,536	195	155	195
**CESC**	16,362	15,113	16,365	534	451	534	4,128	3,890	4,133	193	180	193
**CHOL**	16,379	15,763	16,286	518	499	510	-	-	-	194	188	194
**COAD**	16,374	13,812	16,386	574	505	570	1,216	1,158	1,213	196	185	196
**DLBC**	15,843	13,688	15,786	495	431	500	-	-	-	194	176	178
**ESCA**	16,716	15,024	16,732	515	479	517	-	-	-	193	180	193
**GBM**	16,829	14,520	16,828	-	-	-	4,940	4,609	4,976	191	181	191
**HNSC**	16,653	15,875	16,646	541	533	543	3,829	3,511	3,798	191	180	191
**KICH**	16,392	15,348	16,396	481	462	481	4,090	3,723	4,085	190	178	190
**KIRC**	16,692	13,720	16,686	460	409	461	5,012	4,384	5,008	196	182	191
**KIRP**	16,478	15,965	16,489	480	474	482	4,351	4,115	4,342	197	189	194
**LAML**	-	-	-	-	-	-	-	-	-	-	-	-
**LGG**	16,803	14,110	16,812	553	358	553	5,316	4,557	5,313	193	162	193
**LIHC**	15,855	12,670	15,838	517	419	513	3,395	3,106	3,399	190	185	190
**LUAD**	16,790	15,103	16,790	519	456	518	4,556	4,323	4,583	196	187	196
**LUSC**	16,951	15,706	16,979	525	487	525	4,619	4,251	4,660	196	175	196
**MESO**	16,566	15,638	16,559	531	495	528	-	-	-	190	179	190
**OV**	16,805	15,440	16,808	459	395	460	4,871	4,545	4,861	196	172	196
**PAAD**	17,208	15,920	17,216	538	507	538	-	-	-	197	186	197
**PCPG**	16,409	15,500	16,409	529	512	529	-	-	-	193	193	173
**PRAD**	16,802	14,878	16,798	477	380	477	4,566	4,197	4,571	193	165	193
**READ**	16,464	14,329	16,470	570	492	563	1,242	1,188	1,244	191	190	191
**SARC**	16,219	14,721	16,235	481	424	479	0	0	0	193	179	193
**SKCM**	16,052	15,560	16,057	532	513	531	3,961	3,873	3,991	196	187	196
**STAD**	16,800	15,730	16,735	507	509	512	4,815	4,688	4,838	193	181	193
**TGCT**	17,466	17,477	14,230	670	675	559	-	-	-	194	194	86
**THCA**	16,464	15,111	16,462	513	505	513	4,495	4,171	4,501	194	183	194
**THYM**	16,626	15,691	16,610	615	566	613	-	-	-	194	187	194
**UCEC**	16,659	15,642	16,656	520	498	519	1,335	1,186	1,335	196	187	196
**UCS**	16,947	16,163	16,939	562	532	562	-	-	-	193	152	193
**UVM**	15,584	12,742	15,544	496	440	489	-	-	-	193	193	149
